# The impact of flywheel resistance squat training on lower limb strength in female college basketball players

**DOI:** 10.3389/fphys.2024.1491957

**Published:** 2024-11-25

**Authors:** Lin Xie, Wenhao Qu, Jing Dai, Jiamin Xu, Wenfeng Zhang, Jian Sun, Wenfeng Song, Duanying Li

**Affiliations:** 1 Digitalized Performance Training Laboratory, Guangzhou Sport University, Guangzhou, Guangdong, China; 2 Sports Training Institute, Guangzhou Sport University, Guangzhou, Guangdong, China; 3 Guangdong Provincial Key Laboratory of Human Sports Performance Science, Guangzhou, Guangdong, China

**Keywords:** flywheel resistance squat training, maximal strength, explosive power, female basketball players, jump performance

## Abstract

**Objective:**

This study compared the effects of Flywheel Resistance Squat Training (FRST) vs. Traditional Resistance Squat Training (TRST) on lower body strength in female collegiate basketball players.

**Methods:**

Nineteen participants were randomly assigned to either the FRST group (n = 9) or the TRST group (n = 10) through a random number draw. Both groups underwent a 6-week intervention with training sessions conducted twice a week. The FRST group utilized an inertia of 0.075 kg ·m^2^, while another group trained at 80% of their one-repetition maximum (1RM). Each training session consisted of 4 sets of 8 repetitions with a 3-minute rest between sets. Both groups performed standardized warm-ups and stretches before and after each training session. The effectiveness of the training methods was assessed through the Countermovement Jump (CMJ), Reactive Strength Index (RSI), Eccentric Utilization Ratio, Running Vertical Jump, and 1RM squat.

**Results:**

The FRST and TRST groups showed differences within groups in both CMJ and 1RM squat (*p* < 0.01), with the FRST group demonstrating moderate effect sizes in CMJ (Hedges’ g = 0.59) and 1RM (Hedges’ g = 1.01). However, there were no differences between groups (*p* > 0.05). The Eccentric Utilization Ratio showed a small effect size (*p* = 0.78; $\eta_{p}^{2}=0.01$ ). Additionally, Reactive Strength Index and Running Vertical Jump exhibited low test-retest reliability.

**Conclusion:**

The two groups did not exhibit a statistically significant difference. Nonetheless, both FRST and TRST demonstrated positive effects on 1RM squat and CMJ performance compared to baseline values for each method. Therefore, flywheel resistance training can be considered an effective alternative to traditional resistance training for enhancing strength levels in female basketball players.

## 1 Introduction

Basketball is a high-intensity, contact sport (Tessitore et al., 2006) characterized by frequent transitions between offense and defense and rapid changes in movement (McInnes et al., 1995). This necessitates that players possess advanced neuromuscular capabilities, including power output, strength, and speed (Stojanović et al., 2018). Additionally, players need to react quickly and execute various high-intensity actions, such as sprints, dribbling, jumping, and rapid directional changes, within a short timeframe (Abdelkrim et al., 2010; Alemdaroğlu, 2012). On average, elite athletes, perform 44 ± 7 jumps per game (Abdelkrim et al., 2007) and execute approximately 997 ± 83 sprints and direction changes (Abdelkrim et al., 2007; McInnes et al., 1995). Lower limb strength is essential for executing these technical and tactical movements effectively (Castagna et al., 2007; Alemdaroğlu, 2012) and is one of the most important physical qualities for enhancing performance in high-level basketball players (Ziv and Lidor, 2009).

Resistance training is an effective method for enhancing muscle strength, which can improve lower limb explosive power (Zhang and Zhang, 2023) and maximal strength (Sampson and Groeller, 2016) through increasing neuromuscular adaptation or muscle cross-sectional area. Traditional Resistance Training (TRT) encompasses free weights and weight stacking machines that utilize gravity-dependent loads and is a widely adopted method for resistance training. Although the load/resistance provided by the barbell remains constant throughout the exercise, the specific joint torque required to overcome this resistance varies during t the movement. Particularly, muscle force output may temporarily decrease at specific joint angles (Jorgensen, 1976),and the resistance during the eccentric phase is only 40%–50% of the maximum eccentric load (Hather et al., 1991). The characteristics of barbell training resistance may influence the long-term development of muscle strength and explosive power in athletes (Davies et al., 2016; Kraemer and Fleck, 2007).

Flywheel Resistance Training (FRT) is a method that uses rotating flywheel discs or cones to provide resistance (Beato et al., 2024). During the concentric phase of muscle contraction, pulling a strap connected to the device’s rotational axis causes the flywheel to spin, generating inertial torque and storing kinetic energy, which results in eccentric overload (Kompf and Arandjelović, 2016; Norrbrand et al., 2008) and adaptive resistance (Chiu and Salem, 2006; Berg and Tesch, 1994). This method addresses the issue of insufficient eccentric load in traditional resistance training, enabling participants to achieve improved jumping performance (Allen et al., 2023; Raya-González et al., 2021). Furthermore, a review by Raya-González et al. concluded that FRT may lead to faster adaptations (e.g., strength and power) compared to traditional resistance training programs (Raya-González et al., 2023). As previously mentioned, the transition and fast-break phases in basketball require athletes to perform a series of stretch-shortening cycle (SSC) movements (Komi, 1987). Compared to traditional resistance training, FRT can apply greater load during the eccentric phase of muscle contraction and recruit and activate more motor units (Norrbrand et al., 2010) and generate higher peak force (Norrbrand et al., 2008), thereby enhancing the eccentric phase of SSC (Norrbrand et al., 2011) and showing greater potential to improve lower-limb jump performance in basketball (Stojanović et al., 2021). Additionally, flywheel resistance training shows notable advantages in various phases of periodized training. Specifically, Coratella et al. found that 8 weeks of in-season flywheel training effectively improved soccer players’ jumping performance (Coratella et al., 2019), and other studies have indicated that flywheel resistance training shows benefits in off-season training as well (Stojanović et al., 2021; Younes-Egaña et al., 2023).

Despite these findings, but meta-analysis indicates that the effects of FRT on improving strength and explosive power are similar to those of traditional resistance training (Maroto-Izquierdo et al., 2017; Vicens-Bordas et al., 2018) Furthermore, to date, there is relatively little research on flywheel resistance Training in female athletes, and direct case analyses of the impact of off-season FRT on female athletes are lacking. There remains a lack of consensus regarding whether training experiences designed for male athletes can yield equivalent benefits for female athletes (Abe et al., 2000; Colliander and Tesch, 1991). Therefore, this study aims to investigate the comparative effects in the effects of off-season flywheel resistance squat training (FRST) and traditional resistance squat training (TRST) on lower limb muscle strength in female college basketball players. We hypothesize that flywheel squat training may be more effective than barbell squats in enhancing maximal strength and explosive power.

## 2 Materials and methods

The subject sample size was calculated using G*Power 3.1 software, which determined a minimum total sample size of 18. The calculations were based on an effect size (ES) of 0.5, a significance level (α) of 0.05, and a power of 0.8. To account for an anticipated 10% attrition rate, the study plans to recruit 24 female college basketball athletes. Participants were selected based on the following criteria: (1) university-level female basketball players with at least 3 years of basketball training experience; (2) no physical injuries in the past 6 months; (3) at least 2 years of resistance training experience. Participants were randomly assigned to either the experimental group (FRST, n = 12) or the control group (TRST, n = 12) using a random number generator. During the training period, 5 participants dropped out (2 due to absence exceeding 1 week; 3 due to ankle or arm injuries sustained in competitions outside of the intervention). Ultimately, 19 participants were included in the statistical analysis (age: 20.47 ± 2.27 years; height: 167.26 ± 4.16 cm; weight: 61.56 ± 7.07 kg), with 9 in the experimental group (FRST) and 10 in the control group (TRST) (see Table 1). Throughout the study period, participants were prohibited from engaging in any other high-intensity training, and consumption of alcohol, caffeinated beverages, and other stimulants was restricted. Additionally, a minimum of 48 h was maintained between each experimental session. All research procedures involving human participants adhered to the Declaration of Helsinki. Participants were informed of the study’s purpose and procedures, provided their voluntary consent, and signed an informed consent form. This study was approved by the Ethics Committee for Human Experiments at Guangzhou Sports University (Approval No. 2023LCLL-45) and registered with the Chinese Clinical Trial Registry (Registration No. ChiCTR2400082807).

**TABLE 1:** Basic information of the participates.

Variable	FRST (n = 9)	TRST (n = 10)	*p*-value
Age (year)	20.7 ± 2.0	20.2 ± 2.6	0.66
Height (cm)	167.9 ± 4.7	166.5 ± 3.5	0.50
Body mass (kg)	61.9 ± 4.7	60.1 ± 7.6	0.41

FRST, flywheel resistance squat training; TRST, traditional resistance squat training.

### 2.1 Procedures

The experiment was conducted from April to June 2024. Prior to the experimental intervention, both the experimental and control groups participated in a 2-week familiarization training program consisting of four sessions. Testing was conducted 48 h before the first experimental intervention and 48 h after the last intervention. The assessments included the Countermovement Jump (CMJ), Reactive Strength Index (RSI), Running Vertical Jump, Eccentric Utilization Ratio, and a one-repetition maximal (1RM) barbell back squat test. All tests and experimental interventions were carried out at the same venue under the direct supervision of the same experimenter. The Flywheel Resistance Squat Training (FRST) group and the Traditional Resistance Squat training (TRST) group participated in a 6-week training program. Additionally, Coratella et al. found that following flywheel training, isometric peak torque and muscle soreness returned to values comparable to baseline after 48 and 72 h, respectively (Coratella et al., 2016). Therefore, our experiment will implement interventions on Mondays and Thursdays, occurring twice weekly. The total number of training sessions and the duration of technical and tactical training were consistent for both groups; therefore, we assume that the loading is equivalent between FRST and TRST.

### 2.2 Familiarization

To ensure participants’ familiarity with the content of the program, the following steps were taken in this study: ① to determine the barbell height and belt size for training, participants’ height and waist circumference were measured through interviews and anthropometric methods; ② Participants were provided with a detailed introduction to the standardized operating protocols and technical aspects of flywheel resistance training and barbell squat exercises. To standardize the experimental procedures and ensure consistency across trials.; ③ Subjects were briefed on potential risks, safety procedures, and ethical aspects by signing an informed consent form; ④ During the last familiarization session, tests related to explosive power and maximal strength were conducted, and the load for an 80% 1RM barbell squat was estimated accordingly.

For trainees inexperienced with flywheel resistance training (FRST), research suggests that a minimum of two training sessions is necessary to achieve stability in force production (Sabido et al., 2018). To ensure participants become familiar with the training equipment and master the experimental procedures as well as the correct techniques for applying force during squat training on the flywheel trainer, a total of four familiarization sessions were scheduled.

### 2.3 Outcome measures

Maximal Force Test: Maximal lower-body strength was assessed using the one-repetition maximum (1RM) barbell back squat, in accordance with the protocols outlined in the NSCA’s Guide to Tests and Assessments. Before the formal test, an estimated 1RM was calculated based on the participant’s body weight and training experience. After a general warm-up, participants began with a weight they could comfortably lift for 5 to 10 repetitions. They rested for 1 min before increasing the weight by 10%–20% of their body weight, estimated to allow for 3-5 repetitions. After a 2-minute rest, the weight was increased by another 10%–20%, allow for 2-3 repetitions. Following a 2–4 min rest, the weight was increased by an additional 10%–20%, and participants were asked to perform a 1RM attempt. If successful, the weight was increased by 5%–10% of their body weight after a 2–4 min rest; if unsuccessful, the weight was decreased by 5%–10% and retested. This process continued until a successful 1RM squat was achieved (Miller, 2012).

Explosive Power Test: The assessment comprised CMJ, RSI, Eccentric Utilization Ratio and Running Vertical Jump. CMJ and RSI were measured using the Smart Jump mat from Australia, while the Running Vertical Jump was assessed with a touch-and-go device. For CMJ, participants stood with hands on hips, performed a rapid knee flexion squat to their best angle, and then jumped as high as possible. The Eccentric Utilization Ratio was derived from the ratio of CMJ to SJ (McGuigan et al., 2006), with SJ requiring participants to jump vertically with maximal effort from a knee angle close to 90°, avoiding significant lengthening of the lower limbs. The RSI test required athletes to stand on a 45 cm high box, with hands on hips. After a natural drop of one foot forward, they quickly squatted and jumped as high as possible. The Running Vertical Jump test involved a run-up and jump, with both feet touching the ground prior to takeoff, and the participants’ arms actively swinging. At the peak of the jump, the participant’s fingers brushed the touch-and-go device. All tests required participants to avoid knee flexion, hip extension, or other extraneous movements in the air that could affect the time spent in flight (Jiménez-Reyes et al., 2017). Each explosive force test was conducted twice with 2–3 min intervals, and the best result was used for analysis.

### 2.4 Training routine

Prior to each experimental intervention, participants performed a standardized warm-up based on the RAMP principles outlined by the National Strength and Conditioning Association (NSCA) (Jeffreys, 2006; Jeffreys, 2017). The warm-up consisted of 2 min of foam rolling (targeting the gluteus maximus, quadriceps, hamstrings, tibialis anterior, and gastrocnemius), 3 min of dynamic stretching (including heel raises, cradle stretch, the greatest stretch, and A-skips), 3 min of neural activation (T-rolls, cross steps, and continuous vertical jumps with sprints), and 2 sets of 10 bodyweight squats.

The TRST group performed barbell squats using a free squat rack, with the training intensity set at 80% of their 1RM as recommended by NSCA for effectively improving lower-body maximal strength (Haff and Triplett, 2015). Thus, the TRST group’s training load was 80% of 1RM, comprising 4 sets of 8 repetitions with 3-minute rests between sets. Additionally, to optimally plan the induced stimuli, we presented a comprehensive overview of the training variables (Coratella, 2022) Specifically, ①Range of Movement: full back-squat (0°–140° knee flexion); ②Time Under Tension and Contraction form: During a complete action involving eccentric, isometric, and concentric phases, use a 1–0-1 s tempo; ③focus attention on the entire movement process or task.

Research (Stojanović et al., 2021; Coratella et al., 2019) indicates that an inertia greater than medium can effectively enhance lower-body strength. Additionally, a meta-analysis shows that an inertia moment of 0.075 kg ·m^2^ can produce greater eccentric loads (Sabido et al., 2018) and higher contraction speeds (Piqueras-Sanchiz et al., 2019), positively influencing strength development. Therefore, the FRST group’s training intensity was set at an inertia moment of 0.075 kg ·m^2^, equivalent to 40% of 1RM output power, consisting 4 sets of 8 repetitions with 3-minute rests intervals between sets. During training, participants completed 2 flywheel resistance squats before each set to accelerate the flywheel’s rotation. In each session, participants were required to train with maximum eccentric resistance during the eccentric phase until their hip joints were either lower than or level with their knee joint (Miller, 2012) The Desmotec (Italy) flywheel resistance training equipment was used, which connects via Bluetooth to a client device (Apple or Android) and employs the kMeter software system to monitor training data in real-time. Detailed training content is presented in Table 2.

**TABLE 2:** Training program and training volume.

Training structure	Group	Training method	Sets * reps/Rest interval	Load
Warm-up	FRST and TRST Groups	Foam Rolling Dynamic Stretching Neural Activation 2 sets of 10 Half Squats	10 min	—
Training Intervention	FRST Group	Flywheel Resistance Squat Training	4*8/3 min	0.075 kg ·m^2^
	TRST Group	Traditional Resistance Squat Training	4*8/3 min	80%1RM
Stretching	FRST and TRST Groups	Foam Rolling for Relaxation	10–15 min	—

### 2.5 Statistical analyses

Descriptive statistics are presented as mean ± standard deviation (SD). The level of statistical significance was set at *p* ≤ 0.05. All statistical analyses were conducted using SPSS version 23.0. Normality and homogeneity of variance for all variables were assessed using the Shapiro-Wilk test and the Levene test, respectively. A repeated measures analysis of variance (ANOVA) was employed to evaluate differences between the two groups (FRST vs. TRST), over time (pre-test and post-test, referred to ‘time’) and for the group-by-time interaction effects, with Sidak’s *post hoc* test applied to each outcome measurement. The test-retest reliability of specialized ability tests, such as the Running Vertical Jump was evaluated using coefficients of variation (CV) (Cormack et al., 2008) and intraclass correlation coefficients (ICC) (Koo and Li, 2016) along with a 95% confidence interval (CI), applying a one-way random effects model. Reliability was assessed using a custom spreadsheet (Hopkins, 2006). An ICC value of less than 0.5 indicates poor reliability, between 0.5 and 0.75 indicates moderate reliability, between 0.75 and 0.9 indicates good reliability, and greater than 0.90 indicates excellent reliability. A CV of less than 10% is considered reliable. The effect size of within-group paired differences was estimated using Hedges’ g, with the magnitude of within-group differences categorized as follows: trivial (Hedges’ g ≤ 0.2), small (0.20 < Hedges’ g ≤ 0.60), medium (0.60 < Hedges’ g ≤ 1.20), large (1.20 < Hedges’ g ≤ 2.00), or very large (Hedges’ g ≥ 2.0) (Cohen, 2013). The effect size of the intervention’s group differences was measured using partial eta squared ( $\eta_{p}^{2}$ ), with the degree of group difference categorized as: small $\left(0.01\leq \;\eta_{p}^{2}\leq 0.06\right)$ , medium $\left(0.06\leq \eta_{p}^{2}\;<\;0.14\right)$ , and large $\left(\eta_{p}^{2}\geq 0.14\right)$ (Lachenbruch and Cohen, 1989).

## 3 Results

### 3.1 Muscle strength

**FIGURE 1:**
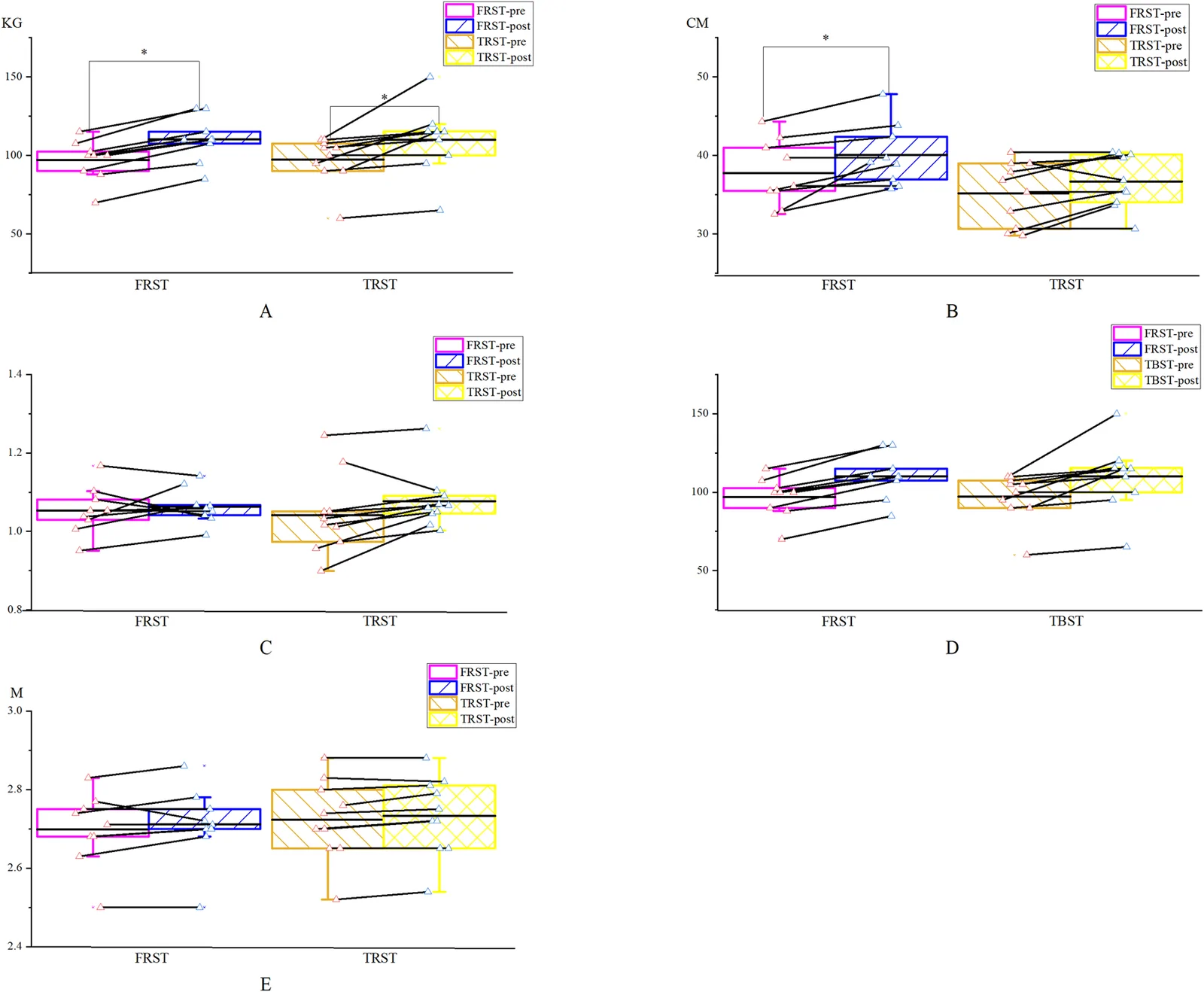
Changes in measured parameters before and after the intervention for the FRST and TBST groups.

The ANOVA results show significant differences within the groups for the 1RM squat. (*p* < 0.001, $\eta_{p}^{2}=0.66$ ; Figures 1–A), but not between groups (*p* = 0.99). Post-hoc comparisons revealed differences between pre- and post-tests for both FRST and TRST (p(FRST) = 0.01; p(TRST) = 0.01), with moderate effect sizes observed (Table 3).

**TABLE 3:** Changes in test indicators before and after experimental intervention in the FRST group and TRST group.

Outcome	Group	Pre	Post	Hedges’ g	Group			Time			Group × time		
					F	p	$\eta_{p}^{2}$	F	p	$\eta_{p}^{2}$	F	p	$\eta_{p}^{2}$
CMJ	FRST	37.74 ± 4.19	40.06 ± 3.97^a^	0.59	2.97	0.10	0.15	15.99	<0.001	0.49	0.81	0.38	0.46
	TRST	35.19 ± 4.05	36.65 ± 3.38	0.41									
EUR	FRST	1.04 ± 0.06	1.08 ± 0.05	0.10	0.08	0.78	0.01	2.55	0.13	0.13	0.76	0.39	0.04
	TRST	1.04 ± 0.08	1.06 ± 0.05	0.03									
RSI	FRST	1.36 ± 0.38	1.35 ± 0.31	−0.03	0.08	0.78	0.01	0.53	0.48	0.03	1.05	0.32	0.06
	TRST	1.34 ± 0.30	1.28 ± 0.23	−0.23									
RVJ	FRST	2.70 ± 0.10	2.71 ± 0.10	0.10	1.16	0.30	0.06	0.55	0.47	0.03	0.95	0.34	0.05
	TRST	2.82 ± 0.32	2.73 ± 0.10	−0.40									
1RM	FRST	97.00 ± 12.99	110.28 ± 14.49^a^	1.01	0	0.99	0.19	33.01	<0.001	0.66	0.01	0.91	0.00
	TRST	97.25 ± 15.11	110.05 ± 21.49^a^	0.72									

^a^ Note: The *post hoc* analyses indicates a significant difference within the group, *p* < 0.05; FRST, flywheel resistance squat training; TRST , traditional resistance squat training; CMJ , countermovement jump; EUR , eccentric utilization ratio; RSI , reactive strength index; RVJ , running vertical jump; 1RM, one-repetition maximum.

### 3.2 Explosive power

The CMJ results show significant differences within groups (*p* < 0.001, $\eta_{p}^{2}=0.49$ ), but not between groups (*p* = 0.10; Figures 1–B). Post-hoc analysis shows significant differences within the FRST group (*p* = 0.02), with a moderate effect size (Hedges’ g = 0.59), whereas another group showed the opposite (*p* = 0.23).

The eccentric utilization ratio (Figures 1–C) and the Reactive Strength Index (Figures 1–D) demonstrated no significant within-group or between-group differences (Table 3). Conversely, the Running Vertical Jump (Figures 1–E) revealed significant within-group differences (*p* = 0.04, $\eta_{p}^{2}=0.22$ ), with no significant between-group differences (*p* = 0.30; Table 3). Reliability measures for the RSI at baseline showed an ICC of 0.60 (95% CI: 0.34–0.80) and a CV of 26.3%. For the two-foot vertical jump test, reliability was reported with an ICC of 0.45 (95% CI: 0.01–0.60) and a CV of 9.0%.

## 4 Discussion

This study, a randomized parallel-controlled trial, aimed to explore the differences between Flywheel Resistance Squat Training and Traditional Resistance Squat Training on lower limb strength in female college basketball players. The results indicate that both training methods showed similar effects in enhancing maximal strength and explosive power. However, FRST may possess potential advantages in certain aspects, although further evidence is needed to fully support this conclusion. Specifically, compared to baseline values, both 1RM squat and countermovement jump exhibited positive trends across groups, with moderate effect sizes. Within the CMJ results, the FRST group showed significant improvement, while another group did not exhibit significant differences. Furthermore, the reliability coefficients for the Relative Strength Index and Relative Vertical Jump were low, preventing a detailed exploration of the effects of these training methods on these metrics.

### 4.1 Muscle strength

Maximal strength is a critical indicator of basketball performance capability, and the 1RM squat is a comprehensive measure of strength levels (Izquierdo et al., 2006). After 6 weeks of FRST and TRST, both groups of female basketball players showed improvement in lower limb maximal strength, with moderate effect sizes within groups and high effect sizes between groups. A recent meta-analysis on flywheel resistance training and traditional resistance strength training conducted on healthy individuals reported similar results. This analysis included ten studies, founding no significant difference between flywheel resistance training and traditional resistance training in enhancing maximal strength (Hu et al., 2024). However, the study indicated that a flywheel resistance training program lasting 12 to 18 sessions can significantly enhance gains compared to traditional resistance training and increase statistical power, as it provides sufficient recovery and over-compensation periods. Therefore, the training cycle will be one of the important factors influencing the effectiveness of flywheel resistance training on maximal strength. Additionally, Bordas et al., ’s 2018 study conducted a study on flywheel resistance training from the perspectives of root mean square electromyography and maximum voluntary contraction (Vicens-Bordas et al., 2018), which also provides physiological supports for our findings.

Several studies confirm that TRST primarily promotes muscle growth and strength increase by increasing load and repetition (Hather et al., 1991; Dudley et al., 1991). In contrast, Fa 6-week FRST study on female soccer players found that flywheel resistance training produced greater overload during the eccentric phase (Pecci et al., 2022), which is advantageous for muscle performance. Furthermore, a systematic review indicates that flywheel resistance training, when properly executed, can produce mechanical eccentric overload, generating greater force during the eccentric phase compared to concentric peak force or power (Beato et al., 2024). FRST’s eccentric overload induces prolonged and high eccentric strain (Douglas et al., 2017), resulting in greater peak tension for the same amount of work, while reducing metabolic cost, thereby maximizing muscle strength, remodeling muscle structure, enhancing motor unit synchronization (Sale, 1988), and stimulating higher electromyographic activity (Moritani and Vries, 1979).

On the other hand, skeletal muscle damage and inflammation can promote muscle protein synthesis and satellite cell differentiation (Chazaud, 2016). Annibalini et al. demonstrated that muscle damage induced by FRST can trigger early molecular responses and systemic muscle adaptations (Annibalini et al., 2019), while altering key markers in the later stages of muscle remodeling and functional adaptation. However, Hakkinen’s research suggests that traditional resistance training can also induce similar muscle adaptations (Häkkinen and Fitness, 1989). Overall, these physiological differences support our findings and highlight the effectiveness of both flywheel and traditional resistance training in enhancing strength in female basketball players.

Nevertheless, a study on amateur football players found that after 6 weeks of intervention, TRST was more effective in increasing squat maximal strength (Sagelv et al., 2020). This discrepancy might be due to differences in maximal neural activation and recovery abilities among athletes of varying skill levels (Petré et al., 2018). Additionally, this study observed that both the experimental and control groups had increasing loads (experimental group: 0.025 kg ·m⁻^2^ to 0.1 kg ·m⁻^2^; control group: 70% 1RM to 85% 1RM). Research indicates that moderate to high inertial moments are most effective in improving maximal strength, which may explain the differences in results.

### 4.2 Explosive force

Specialized basketball techniques (e.g., critical rebounds, blocks, and dunks) are closely related to lower limb explosive power (Abdelkrim et al., 2010; Alemdaroğlu, 2012) This study selected CMJ, RSI, Reactive Strength Index and Eccentric Utilization Ratio as direct variables to assess the impact of FRST and TRST on explosive power. After 6 weeks of intervention, results showed no significant differences between FRST and TRST in improving CMJ scores for female basketball players. However, both methods exhibited varying degrees of improvement compared to baseline, with the FRST group showing significant within-group differences. This finding aligns with Coratella et al.'s previous research (Coratella et al., 2019), which found that both FRST and TRST improved CMJ performance in football players, although no significant between-group differences were observed. Additionally, a 6-week study on female football players (Pecci et al., 2022) found no significant effects of either training method on CMJ scores, possibly due to a lack of high-intensity jumping in their training. However, Fernandez et al.'s 6-week study (Gonzalo et al., 2014) indicated that FRST improved CMJ performance in both male and female subjects, suggesting that higher training frequency and longer training periods may be more beneficial for improving CMJ scores in female athletes. In contrast, Wang Jiaoqin et al.'s research (Wang et al., 2024) found a significant improvement in CMJ scores for female volleyball players after 8 weeks of intervention (*p* = 0.04). Thus, training frequency, cycle length, and the types of exercises selected may contribute to discrepancies between this study’s results and those of other studies. A meta-analysis (Hu et al., 2024) also supports this view, indicating that the choice of training program significantly impacts research outcomes.

Gonzalo et al. proposed that FRST can activate more cross-bridges, generating higher levels of force during the eccentric phase of the stretch-shortening cycle (SSC) in CMJ (Gonzalo-Skok et al., 2017). This mechanism may lead to improved SSC performance. Additionally, increased threshold excitability of the Golgi tendon organs (McNeely and Sandler, 2007) and changes in neuromuscular coordination may contribute to the improvement in CMJ performance among female basketball players. However, other studies suggest that traditional resistance training, by enhancing muscle and tendon elasticity, can achieve similar changes (Häkkinen and Fitness, 1989).

The Eccentric Utilization Ratio has been validated as a useful indicator for assessing elite athletes’ training status and their utilization of the stretch-shortening cycle (Hawkins et al., 2009). After 6 weeks of intervention, the ratio showed no statistical differences either within or between groups, but both training methods demonstrated positive effects. A value of 1.1 not only reflects excellent explosive performance but is also an ideal standard sought by coaches (Balsom, 1994). Despite the absence of significant differences in Eccentric Utilization Ratio between the groups, the FRST group was higher than another, approaching the optimal value of 1.1. Thus, FRST may be more effective in improving this metric. Additionally, a study involving 142 team athletes indicated that the Eccentric Utilization Ratio based on height before and after the season showed results consistent with those observed in this study (McGuigan et al., 2006). Another study (Martinez-Aranda and Fernandez-Gonzalo, 2017) suggests that FRST can enhance the ability to repeatedly perform high-intensity eccentric actions following a period of training, leading to improvements in the Eccentric Utilization Ratio. The Reactive Strength Index measures an athlete’s reactive jumping ability and ability to handle impact forces (Jarvis et al., 2022). A meta-analysis on flywheel resistance training showed results differing from this study (Buonsenso et al., 2023), indicating positive results for RSI in most samples with different training programs. This discrepancy might be due to the short experimental period, which may not reach the threshold required to cause changes in RSI. However, the low Reliability measures for the Reactive Strength Index and the Running Vertical Jump in this study may have introduced errors in the analysis. Therefore, this study could not explore the impact of flywheel resistance training on specialized explosive power. This might be due to the complex nature of these jump modes, which require a combination of factors such as ground force application, proper force distribution, rhythm changes, and core stability. Future research should consider these factors comprehensively.

## 5 Conclusion

Both flywheel resistance squat training and traditional resistance squat training effectively enhance lower limb strength and explosive power in female basketball players over a 6-week period. Specifically, although no significant differences were observed between the two training methods in terms of One-Repetition Maximum, Countermovement Jump and Eccentric Utilization Ratio, both methods demonstrated positive improvements compared to baseline values. The flywheel resistance training group, in particular, showed a higher effect size in improving CMJ scores, suggesting its potential advantage. Therefore, coaches may incorporate flywheel resistance training as an effective alternative to traditional resistance training into their programs to enhance athletes’ jumping ability and optimize training outcomes.

## 6 Limitations and prospects

1. The training load used in this study only involves high moment of inertia. It is recommended to add a low moment f inertia experimental group in future research to compare and analyze the differences in training effects under different moment of inertia conditions. In addition, considering the short intervention period, it is recommended to extend the training period and adjust the training intensity according to the cyclical arrangement of the basketball season to meet the actual needs of basketball season training, further exploring the application effect of flywheel training in basketball season.
2. Given that the athletes participating in this study have specific competitive levels, the generalizability of the research results may be limited and only applicable to athletes with similar competitive levels. Considering the important role of basketball players’ upper limbs in confrontation and shooting stability, it is recommended to add upper limb flywheel training to future training programs.
3. The fixed load used in the experimental design limits the in-depth exploration of the optimal adaptive response. Although this study assumes that the loads of the two training schemes match, the differences in the working mechanisms of the barbell and flywheel may lead to differences in actual resistance, which should be considered in future research.
4. This study failed to consider the potential impact of menstrual cycle on the training status and motivation of participants. It is recommended to include this variable in future experimental intervention designs, especially during the testing phase, to more comprehensively evaluate the training effectiveness.

## Data Availability

The datasets presented in this study can be found in online repositories. The names of the repository/repositories and accession number(s) can be found in the article/Supplementary Material.

## References

[B1] Abdelkrim N. B. Chaouachi A. Chamari K. Chtara M. Castagna C. (2010). Positional role and competitive-level differences in elite-level men's basketball players. J. Strength Cond. Res. 24, 1346–1355. 10.1519/SC.0b013e3181cf7510 20393355

[B2] Abdelkrim N. B. El Fazaa S. El Ati J. (2007). Time–motion analysis and physiological data of elite under-19-year-old basketball players during competition. Br. J. Sports Med. 41, 69–75. 10.1136/bjsm.2006.032318 17138630 PMC2658931

[B3] Abe T. Dehoyos D. V. Pollock M. L. Garzarella L. J. E. J. O. A. P. (2000). Time course for strength and muscle thickness changes following upper and lower body resistance training in men and women. Eur. J. Appl. physiology 81, 174–180. 10.1007/s004210050027 10638374

[B4] Alemdaroğlu U. (2012). The relationship between muscle strength, anaerobic performance, agility, sprint ability and vertical jump performance in professional basketball players. J. Hum. Kinet. 31, 149–158. 10.2478/v10078-012-0016-6 23486566 PMC3588656

[B5] Allen W. J. De Keijzer K. L. Raya-GonzáLEZ J. Castillo D. Coratella G. Beato M. J. R. I. S. M. (2023). Chronic effects of flywheel training on physical capacities in soccer players: a systematic review. A Syst. Rev. 31, 228–248. 10.1080/15438627.2021.1958813 34315310

[B6] Annibalini G. Contarelli S. Lucertini F. Guescini M. Maggio S. Ceccaroli P. (2019). Muscle and systemic molecular responses to a single flywheel based iso-inertial training session in resistance-trained men. Front. Physiology 10, 554. 10.3389/fphys.2019.00554 31143128 PMC6521220

[B7] Balsom P. (1994). Evaluation of physical performance, 111–116.

[B8] Beato M. De Keijzer K. L. MuñOZ-Lopez A. Raya-GonzáLEZ J. Pozzo M. Alkner B. A. (2024). Current guidelines for the implementation of flywheel resistance training technology in sports: a consensus statement. Sports Med. 54, 541–556. 10.1007/s40279-023-01979-x 38175461 PMC10978721

[B9] Berg H. E. Tesch A. (1994). A gravity-independent ergometer to be used for resistance training in space. Aviat. space, Environ. Med. 65, 752–756. 7980338

[B10] Buonsenso A. Centorbi M. Iuliano E. Di Martino G. Della Valle C. Fiorilli G. (2023). A systematic review of flywheel training effectiveness and application on sport specific performances. Sports 11, 76. 10.3390/sports11040076 37104150 PMC10144427

[B11] Castagna C. Manzi V. D'Ottavio S. Annino G. Padua E. Bishop D. (2007). Relation between maximal aerobic power and the ability to repeat sprints in young basketball players. J. Strength Cond. Res. 21, 1172–1176. 10.1519/R-20376.1 18076232

[B12] Chazaud B. (2016). Inflammation during skeletal muscle regeneration and tissue remodeling: application to exercise-induced muscle damage management. Immunol. Cell Biol. 94, 140–145. 10.1038/icb.2015.97 26526620

[B13] Chiu L. Z. Salem G. J. (2006). Comparison of joint kinetics during free weight and flywheel resistance exercise. J. Strength Cond. Res. 20, 555–562. 10.1519/R-18245.1 16937968

[B14] Cohen J. (2013). Statistical power analysis for the behavioral sciences. Routledge.

[B15] Colliander E. Tesch P. (1991). Responses to eccentric and concentric resistance training in females and males. Acta physiol. Scand. 141, 149–156. 10.1111/j.1748-1716.1991.tb09063.x 2048403

[B16] Coratella G. (2022). Appropriate reporting of exercise variables in resistance training protocols: much more than load and number of repetitions. Sports Med. Open 8, 99. 10.1186/s40798-022-00492-1 35907047 PMC9339067

[B17] Coratella G. Beato M. Cè E. Scurati R. Milanese C. Schena F. (2019). Effects of in-season enhanced negative work-based vs traditional weight training on change of direction and hamstrings-to-quadriceps ratio in soccer players. Biol. Sport 36, 241–248. 10.5114/biolsport.2019.87045 31624418 PMC6786325

[B18] Coratella G. Chemello A. Schena F. Fitness P. (2016). Muscle damage and repeated bout effect induced by enhanced eccentric squats. J. Sports Med. Phys. Fit. 56, 1540–1546. 10.1016/j.jde.2008.06.030 26671347

[B19] Cormack S. J. Newton R. U. Mcguigan M. R. Doyle T. L. (2008). Reliability of measures obtained during single and repeated countermovement jumps. Int. J. sports physiology Perform. 3, 131–144. 10.1123/ijspp.3.2.131 19208922

[B20] Davies T. Orr R. Halaki M. Hackett D. (2016). Effect of training leading to repetition failure on muscular strength: a systematic review and meta-analysis. Sports Med. 46, 487–502. 10.1007/s40279-015-0451-3 26666744

[B21] Douglas J. Pearson S. Ross A. Mcguigan M. R. (2017). Eccentric exercise: physiological characteristics and acute responses. Sports Med. 47, 663–675. 10.1007/s40279-016-0624-8 27638040

[B22] Dudley G. A. Tesch P. Miller B. Buchanan P. (1991). Importance of eccentric actions in performance adaptations to resistance training. Aviat. Space, Environ. Med. 62, 543–550. 1859341

[B23] Gonzalo R. Lundberg T. R. Alvarez-Alvarez L. De Paz J. A. (2014). Muscle damage responses and adaptations to eccentric-overload resistance exercise in men and women. Eur. J. Appl. physiology 114, 1075–1084. 10.1007/s00421-014-2836-7 24519446

[B24] Gonzalo-Skok O. Tous-Fajardo J. Valero-Campo C. Berzosa C. Bataller A. V. Arjol-Serrano J. L. (2017). Eccentric-overload training in team-sport functional performance: constant bilateral vertical versus variable unilateral multidirectional movements. Int. J. Sports Physiology Perform. 12, 951–958. 10.1123/ijspp.2016-0251 27967273

[B25] Haff G. G. Triplett N. T. (2015). Essentials of strength training and conditioning. 4th edition. Human kinetics.

[B26] HäKKINEN K. Fitness P. (1989). Neuromuscular and hormonal adaptations during strength and power training. A Rev. 29, 9–26. 2671501

[B27] Hather B. Tesch P. Buchanan P. Dudley G. (1991). Influence of eccentric actions on skeletal muscle adaptations to resistance training. Acta Physiol. Scand. 143, 177–185. 10.1111/j.1748-1716.1991.tb09219.x 1835816

[B29] Hawkins S. B. Doyle T. L. Mcguigan M. R. (2009). The effect of different training programs on eccentric energy utilization in college-aged males. J. Strength Cond. Res. 23, 1996–2002. 10.1519/JSC.0b013e3181b3dd57 19855323

[B30] Hopkins W. G. (2006). Spreadsheets for analysis of controlled trials, with adjustment for a subject characteristic. Sportscience 10, 46–50.

[B31] Hu Z. Liu Y. Huang K. Huang H. Li F. Yuan X. J. L. (2024). Comparing the effect of isoinertial flywheel training and traditional resistance training on maximal strength and muscle power in healthy people: a systematic review and meta-analysis. Life (Basel), 14 **,** 908, 10.3390/life14070908 39063661 PMC11277740

[B32] Izquierdo M. Ibanez J. González-Badillo J. J. Häkkinen K. Ratamess N. A. Kraemer W. J. (2006). Differential effects of strength training leading to failure versus not to failure on hormonal responses, strength, and muscle power gains. J. Appl. physiology 100, 1647–1656. 10.1152/japplphysiol.01400.2005 16410373

[B33] Jarvis P. Turner A. Read P. Bishop C. (2022). Reactive strength index and its associations with measures of physical and sports performance: a systematic review with meta-analysis. Sports Med. 52, 301–330. 10.1007/s40279-021-01566-y 34606061

[B34] Jeffreys I. (2006). Warm up revisited–the “ramp” method of optimising performance preparation. J Uksca J. 6, 15–19.

[B35] Jeffreys I. (2017). RAMP warm-ups: more than simply short-term preparation. Prof. Strength Cond. 44, 17–23.

[B36] Jiménez-Reyes P. Samozino P. Pareja-Blanco F. Conceição F. Cuadrado-Peñafiel V. González-Badillo J. J. (2017). Validity of a simple method for measuring force-velocity-power profile in countermovement jump. Int. J. Sports Physiology Perform. 12, 36–43. 10.1123/ijspp.2015-0484 27002490

[B37] Jorgensen K. (1976). Force-velocity relationship in human elbow flexors and extensors. Biomech. va, 145–151.

[B38] Komi P. V. (1987). “Neuromuscular factors related to physical performance,” in Muscular function in exercise and training (Karger Publishers).

[B39] Kompf J. Arandjelović O. (2016). Understanding and overcoming the sticking point in resistance exercise. Sports Med. 46, 751–762. 10.1007/s40279-015-0460-2 26758462 PMC4887540

[B40] Koo T. K. Li M. Y. (2016). A guideline of selecting and reporting intraclass correlation coefficients for reliability research. J. Chiropr. Med. 15, 155–163. 10.1016/j.jcm.2016.02.012 27330520 PMC4913118

[B41] Kraemer W. J. Fleck S. J. (2007). Optimizing strength training: designing nonlinear periodization workouts. Human Kinetics.

[B42] Lachenbruch P. A. Cohen J. (1989). Statistical power analysis for the behavioral Sciences (2nd ed.). J. Am. Stat. Assoc. 84, 1096–1097. 10.2307/2290095

[B43] Maroto-Izquierdo S. GarcíA-López D. Fernandez-Gonzalo R. Moreira O. C. González-Gallego J. De Paz J. A. (2017). Skeletal muscle functional and structural adaptations after eccentric overload flywheel resistance training: a systematic review and meta-analysis. J. Sci. Med. sport 20, 943–951. 10.1016/j.jsams.2017.03.004 28385560

[B44] Martinez-Aranda L. M. Fernandez-Gonzalo R. (2017). Effects of inertial setting on power, force, work, and eccentric overload during flywheel resistance exercise in women and men. J. Strength Cond. Res. 31, 1653–1661. 10.1519/JSC.0000000000001635 28538317

[B45] Mcguigan M. R. Doyle T. L. Newton M. Edwards D. J. Nimphius S. Newton R. U. (2006). Eccentric utilization ratio: effect of sport and phase of training. J. Strength Cond. Res. 20, 992–995. 10.1519/R-19165.1 17194252

[B46] Mcinnes S. Carlson J. Jones C. Mckenna M. (1995). The physiological load imposed on basketball players during competition. J. Sports Sci. 13, 387–397. 10.1080/02640419508732254 8558625

[B47] Mcneely E. Sandler D. (2007). Power plyometrics: the complete program. Meyer and Meyer Verlag.

[B48] Miller T. A. (2012). NSCA's Guide to tests and assessments. Human Kinetics.

[B49] Moritani T. Vries H. A. (1979). Neural factors versus hypertrophy in the time course of muscle strength gain. Am. J. Phys. Med. 58, 115–130. 453338

[B50] Norrbrand L. Fluckey J. D. Pozzo M. Tesch P. A. (2008). Resistance training using eccentric overload induces early adaptations in skeletal muscle size. Eur. J. Appl. Physiol. 102, 271–281. 10.1007/s00421-007-0583-8 17926060

[B51] Norrbrand L. Pozzo M. Tesch P. (2010). Flywheel resistance training calls for greater eccentric muscle activation than weight training. Eur. J. Appl. Physiol. 110, 997–1005. 10.1007/s00421-010-1575-7 20676897

[B52] Norrbrand L. Tous-Fajardo J. Vargas R. Tesch P. A. J. A. Medicine E. (2011). Quadriceps muscle use in the flywheel and barbell squat. Aviat. Space Environ. Med. 82, 13–19. 10.3357/asem.2867.2011 21235100

[B53] Pecci J. Muñoz-López A. Jones P. Sañudo B. (2022). Effects of 6 weeks in-season flywheel squat resistance training on strength, vertical jump, change of direction and sprint performance in professional female soccer players. Biol. Sport 40, 521–529. 10.5114/biolsport.2023.118022 37077802 PMC10108773

[B54] Petré H. WernståL F. Mattsson C. M. (2018). Effects of flywheel training on strength-related variables: a meta-analysis. Sports medicine-open 4, 55. 10.1186/s40798-018-0169-5 30547232 PMC6292829

[B55] Piqueras-Sanchiz F. Martin-Rodriguez S. MartíNEZ-Aranda L. M. Lopes T. R. Raya-Gonzalez J. Garcia-Garcia O. (2019). Effects of moderate vs. high iso-inertial loads on power, velocity, work and hamstring contractile function after flywheel resistance exercise. PLoS One 14, e0211700. 10.1371/journal.pone.0211700 30730959 PMC6366769

[B56] Raya-González J. Castillo D. Beato M. J. S. Journal C. (2021). The flywheel paradigm in team sports: a soccer approach. Strength and Cond. J. 43 **,** 12–22. 10.1519/ssc.0000000000000561

[B57] Raya-González J. Castillo D. De Keijzer K. L. Beato M. J. S. Journal C. (2023). Considerations to optimize strength and muscle mass gains through flywheel resistance devices: a narrative review. Strength Cond. J. 45 **,** 111–121. 10.1519/SSC.0000000000000732

[B58] Sabido R. Hernández-Davó J. L. Pereyra-Gerber G. T. (2018). Influence of different inertial loads on basic training variables during the flywheel squat exercise. Int. J. Sports Physiol. Perform. 13, 482–489. 10.1123/ijspp.2017-0282 28872379

[B59] Sagelv E. H. Pedersen S. Nilsen L. P. R. Casolo A. Welde B. Randers M. B. (2020). Flywheel squats versus free weight high load squats for improving high velocity movements in football. A randomized controlled trial. BMC Sports Sci. Med. Rehabilitation 12, 61. 10.1186/s13102-020-00210-y 33024564 PMC7532637

[B60] Sale D. G. (1988). Neural adaptation to resistance training. Med. Sci. sports Exerc. 20, S135–S145. 10.1249/00005768-198810001-00009 3057313

[B61] Sampson J. A. Groeller H. (2016). Is repetition failure critical for the development of muscle hypertrophy and strength? Scand. J. Med. Sci. sports 26, 375–383. 10.1111/sms.12445 25809472

[B62] Stojanović M. D. Mikić M. Drid P. Calleja-GonzáLEZ J. Maksimović N. Belegišanin B. (2021). Greater power but not strength gains using flywheel versus equivolumed traditional strength training in junior basketball players. Int. J. Environ. Res. 18, 1181. 10.3390/ijerph18031181 33572738 PMC7908554

[B63] Stojanović E. Stojiljković N. Scanlan A. T. Dalbo V. J. Berkelmans D. M. Milanović Z. J. S. M. (2018). The activity demands and physiological responses encountered during basketball match-play: a systematic review. Sports Med. 48, 111–135. 10.1007/s40279-017-0794-z 29039018

[B64] Tessitore A. Tiberi M. Cortis C. Rapisarda E. Meeusen R. Capranica L. (2006). Aerobic-anaerobic profiles, heart rate and match analysis in old basketball players. Gerontology 52, 214–222. 10.1159/000093653 16849864

[B65] Vicens-Bordas J. Esteve E. Fort-Vanmeerhaeghe A. Bandholm T. Thorborg K. (2018). Is inertial flywheel resistance training superior to gravity-dependent resistance training in improving muscle strength? A systematic review with meta-analyses. J. Sci. Med. Sport 21, 75–83. 10.1016/j.jsams.2017.10.006 29107539

[B66] Wang J. Zhang Q. Chen W. Fu H. Zhang M. Fan Y. (2024). The effect of flywheel complex training with eccentric-overload on muscular adaptation in elite female volleyball players. PeerJ 12, e17079. 10.7717/peerj.17079 38525282 PMC10961060

[B67] Younes-EgañA O. Bird S. P. Calleja-GonzáLEZ J. (2023). From theory to practice: a worldwide cross-sectional survey about flywheel training in basketball. Int. J. Sports Physiology Perform. 19, 185–194. 10.1123/ijspp.2023-0202 38134893

[B68] Zhang S. Zhang Z. (2023). Strength training and physical improvement in basketball. Rev. Bras. Med. do Esporte 29, e2022. 10.1590/1517-8692202329012022_0532

[B69] Ziv G. Lidor R. (2009). Physical attributes, physiological characteristics, on-court performances and nutritional strategies of female and male basketball players. Sports Med. 39, 547–568. 10.2165/00007256-200939070-00003 19530751

