# Correction: The impact of flywheel resistance squat training on lower limb strength in female college basketball players

**DOI:** 10.3389/fphys.2026.1996989

**Published:** 2026-10-06

**Authors:** Lin Xie, Wenhao Qu, Jing Dai, Jiamin Xu, Wenfeng Zhang, Jian Sun, Wenfeng Song, Duanying Li

**Affiliations:** 1 Digitalized Performance Training Laboratory, Guangzhou Sport University, Guangzhou, Guangdong, China; 2 Sports Training Institute, Guangzhou Sport University, Guangzhou, Guangdong, China; 3 Guangdong Provincial Key Laboratory of Human Sports Performance Science, Guangzhou, Guangdong, China

**Keywords:** flywheel resistance squat training, maximal strength, explosive power, female basketball players, jump performance

To avoid potential ambiguity regarding the timing of the study, the description of the experimental period has been clarified, as the original statement referred to the initially planned period rather than the period during which the experiment was actually conducted.

A correction has been made to section **2 Materials and methods,** *2.1 Procedures*, paragraph 1. The correct text reads: “The experiment was conducted from April to June 2024.”

The original version of this article has been updated.

