# Supplementary material for: The impact of flywheel resistance squat training on lower limb strength in female college basketball players
Source: Front Physiol. 2024 Nov 25;15:1491957. doi: 10.3389/fphys.2024.1491957 (PMC11625767; doi:10.3389/fphys.2024.1491957)
Supplement: Supplementary file 1 [file Table1.docx]

Supplementary Material

# Supplementary Figures and Tables


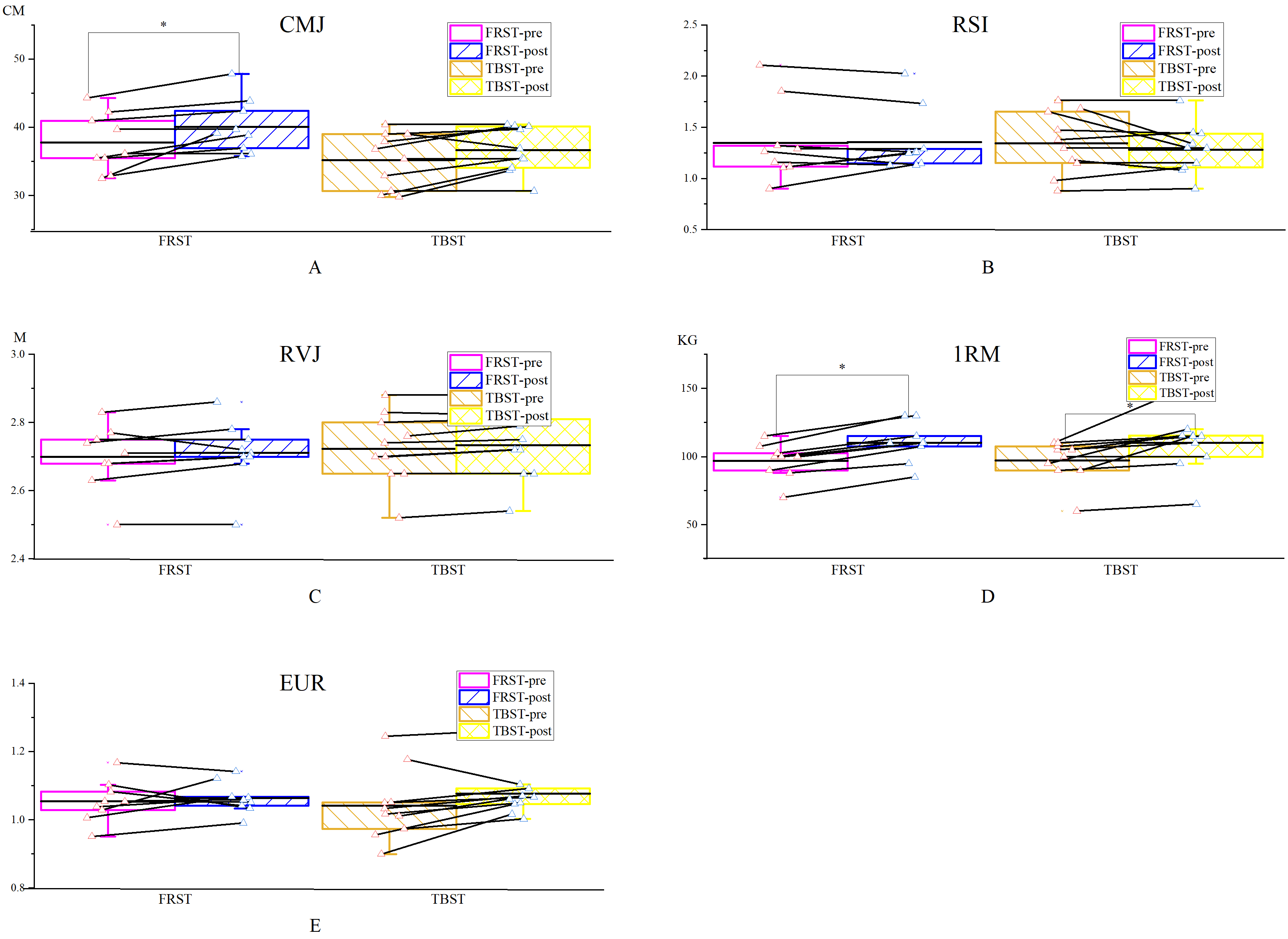
Figure 1. Changes in measured parameters before and after the intervention for the FRST and TBST groups.
